# Cardiac function, heart failure indicators and inflammatory factors in continuous mild hypothermic hemodialysis patients after valvular heart disease surgery

**DOI:** 10.5937/jomb0-60316

**Published:** 2026-03-17

**Authors:** Mao Yun, Zhu Li, Chen Huilan, Li Danxin, Yuan Chikai, Wang Renqiang, Liu Jian

**Affiliations:** 1 Jinhua Hospital Affiliated to Zhejiang University School of Medicine, Department of Geriatrics, Jinhua City, China; 2 Jinhua Hospital Affiliated to Zhejiang University School of Medicine, Intensive Care Unit, Jinhua City, China; 3 People's Hospital of Wuhan University, Department of Cardiology, Wuhan, China; 4 The Affiliated Dazu's Hospital of Chongqing Medical University (The People's Hospital of Dazu, Chongqing, Cardiothoracic Surgery, Tangxiang Sub-district, Dazu District, Chongqing City, China

**Keywords:** continuous mild hypothermic hemodialysis, valvular heart disease surgery, inflammatory factors, cardiac function, heart failure indicators, kontinuirana hemodijaliza u blagoj hipotermiji, operacija bolesti srčanih zalistaka, inflamatorni faktori, srčana funkcija, pokazatelji srčane insuficijencije

## Abstract

**Background:**

To compare the effects of continuous hemodialysis at normal temperature and mild hypothermia in the treatment of cardiogenic shock after valvular heart disease surgery and its influences on patients' cardiac function, heart failure indicators and inflammatory factors.

**Methods:**

After surgery for valvular heart disease, 180 patients with cardiogenic shock were brought to our hospital between January 2023 and December 2024. Patients in the control group received continuous hemodialysis treatment at normal temperature, whereas patients in the observation group received continuous hemodialysis treatment at mild hypothermia. The postoperative drainage volume, blood purification time, ventilator assistance time, ICU stay time, and occurrence of death, arrhythmia and infection were compared between the two groups. Cardiac function indicators, laboratory indicators of heart failure, and serum creatinine (Cr) and inflammatory factor data were compared between the two patient groups before and after therapy.

**Results:**

Postoperative drainage volume, ventilator assistance time, intensive care unit (ICU) stay, blood purification time and mortality rate (P&lt;0.05) were analysed. The laboratory measurements of BNP , hs-CRP , and CR-related heart failure did not differ before treatment (P&gt;0.05). BNP , hs-CRP , and CR-related heart failure were laboratory markers that were reduced in both groups after treatment; the levels in the observation group were lower than those in the control group (P&lt;0.05). Tumour necrosis factor-α (TNF-α), interleukin-6 (IL-6), and interleukin-1 (IL-1) were present at the same pretreatment levels in both groups (P&gt;0.05). The levels decreased after treatment and were lower in the observation group than in the control group (P&lt;0.05).

**Conclusions:**

For patients with cardiogenic shock after valvular heart disease surgery, mild hypothermia hemodialysis treatment can further reduce the postoperative drainage volume and hemopurification time, promote early recovery, and reduce the mortality rate. Moreover, mild hypothermia hemodialysis treatment can further improve the cardiac function of patients and alleviate heart failure. The levels of inflammatory factors in the patient's body should be reduced.

## Abstract (sr)

**Uvod:**

Upoređivanje efekata kontinuirane hemodijalize na normalnoj temperaturi i u uslovima blage hipotermije u lečenju kardiogenog šoka nakon operacije bolesti srčanih zalistaka, kao i procena njihovog uticaja na srčanu funkciju, pokazatelje srčane insuficijencije i inflamatorne faktore.

**Metode:**

Između januara 2023. i decembra 2024. godine, u našoj ustanovi je lečeno ukupno 180 pacijenata koji su razvili kardiogeni šok nakon operacije bolesti srčanih zalistaka. Pacijenti u kontrolnoj grupi su bili na kontinuiranoj hemodijalizi na normalnoj temperaturi, dok je kod pacijenata u posmatranoj grupi primenjivana kontinuirana hemodijaliza u uslovima blage hipotermije. Upoređivani su postoperativna količina drenaže, vreme hemopurifikacije, trajanje mehaničke ventilacije, dužina boravka u jedinici intenzivne nege (JIN), kao i učestalost smrtnog ishoda, aritmija i infekcija. Takođe su analizirani parametri srčane funkcije, laboratorijski pokazatelji srčane insuficijencije, serumski kreatinin (Cr) i nivoi inflamatornih faktora, pre i posle terapije.

**Rezultati:**

Posmatrana grupa je imala značajno manju postoperativnu drenažu, kraće trajanje mehaničke ventilacije, skraćen boravak u JIN, kraće vreme hemopurifikacije i nižu stopu mortaliteta u poređenju sa kontrolnom grupom (P&lt;0,05). Početni nivoi BNP hs-CRP i drugih markera srčane insuficijencije nisu se razlikovali između grupa (P&gt;0,05). Nakon terapije, ovi markeri su opali u obe grupe, pri čemu je pad bio izraženiji u posmatranoj grupi (P&lt;0,05). Slično, osnovni nivoi TNF-α, IL-6 i IL-1 nisu se razlikovali među grupama (P&gt;0,05), ali su nakon lečenja značajno opali u obe grupe, sa nižim vrednostima u posmatranoj grupi (P&lt;0,05).

**Zaključak:**

Kod pacijenata sa kardiogenim šokom nakon operacije bolesti srčanih zalistaka, kontinuirana hemodijaliza u uslovima blage hipotermije pokazuje prednosti u odnosu na normotermnu hemodijalizu. Smanjuje postoperativnu drenažu i vreme hemopurifikacije, ubrzava oporavak i snižava mortalitet. Pored toga, poboljšava srčanu funkciju, ublažava insuficijenciju i smanjuje sistemski inflamatorni odgovor.

## Introduction

With increasing population ageing, the incidence of senile valvular disease and valvular lesions caused by coronary heart disease and myocardial infarction is gradually increasing [1]. Surgical treatment is a common therapy for heart valve diseases, with marked effects and the ability to improve the clinical symptoms of patients. Cardiogenic shock, a common perioperative complication of heart valve disease, often manifests as poor tissue perfusion, cold limbs, hypotension, internal environment disorders, soft acidosis, etc. If not treated in time, it can lead to multiple organ dysfunction syndrome and other conditions, with a relatively high mortality rate [2][3]. Currently, in clinical practice, continuous hemodialysis is mainly recommended for patients with cardiogenic shock after heart valve disease surgery to correct the imbalance of blood supply in the body and reduce the mortality rate of patients. Although the effect of continuous hemodialysis in treating cardiogenic shock after heart valve disease surgery has gradually been recognised by many medical professionals and patients, there are still some controversies regarding the application of the temperature of the replacement fluid [4][5][6]. Hypothermia therapy can further reduce oxygen consumption, and through mild hypothermia treatment, it can reduce myocardial damage caused by myocardial ischemia after cardiac valve surgery and resuscitation and improve the cardiac function and inflammatory response of patients. Another study [7] suggested that continuous hemodialysis treatment at a normal temperature can reduce the stress response caused by treatment in patients.

Patients who have undergone valvular heart disease surgery often face the risk of cardiac dysfunction and heart failure, especially during hemodialysis. In recent years, continuous mild hypothermic hemodialysis, as a new treatment approach, has gradually attracted attention. Continuous mild hypothermic hemodialysis may help reduce the burden on the heart and improve cardiac function by lowering body temperature and slowing the metabolic rate. However, its actual effect on patients after valvular heart disease surgery has not been thoroughly studied. 

This study explored the effects of continuous mild hypothermic hemodialysis on cardiac function, heart failure indicators and inflammatory factors in patients after valvular heart disease surgery. Systematic evaluation of changes in cardiac function, heart failure indicators such as N-terminal pro-brain natriuretic peptide (NT-proBNP), and the levels of inflammatory factors is needed. Although continuous mild hypothermic hemodialysis has potential advantages, it still faces many challenges in practical operation, such as the risk of complications caused by hypothermic changes in blood circulation and the impact of individual patient differences on treatment outcomes. Therefore, this study not only helps to verify the effectiveness of this treatment method but also provides valuable reference data for its clinical application.

Therefore, to further improve the clinical efficacy of patients with cardiogenic shock after valvular heart disease surgery, this study compared the effects of continuous hemodialysis under normal and mild hypothermia on patients’ cardiac function, heart failure indicators, and inflammatory factors.

## Materials and methods

### Analysis of clinical case data

The study involved 180 patients who were brought to our hospital between January 2023 and December 2024 with cardiogenic shock following surgery for valvular heart disease. Two groups of 90 patients each were created from these patients: an observation group and a control group. The general data for the two patient groups did not differ, as shown in Table 1 (P>0.05).

**Table 1:** Analysis of clinical case data.

Groups	n	Gender<br>(Male/Female)	Age<br>(Years)	Heart to<br>chest ratio	Surgical type (n)		
					Double valve<br>replacement surgery	Aortic valve<br>replacement	Mitral valve<br>replacement
Observation group	90	46/44	47.27±4.26	0.64±0.13	36	34	20
Control group	90	50/40	47.21±3.21	0.66±0.14	32	40	18
x^2^/t	–	0.519	0.519	0.295	–	0.268	–
P	–	0.597	0.597	0.745	–	0.605	–

### Inclusion and exclusion criteria

The following were the requirements for patient inclusion: fulfilled the criteria for valvular heart disease diagnosis [8] and chose to undergo elective open-heart surgery under external circulation. Cardiogenic shock occurred after the operation [9], CI<2.5 L/(min·m^2^), diastolic blood pressure <60 mm Hg and systolic blood pressure <90 mm Hg; the patient met the indications for blood purification treatment, was aged ≥18 years, and provided informed consent.

The exclusion criteria were patients who had undergone secondary surgical trauma or perioperative myocardial ischemia after surgery, those with concurrent malignant tumours, and those with mental disorders who could not cooperate with researchers.

### Research methods

All patients were indwelled with radial artery manometry tubes, three-lumen venous tubes, five-lumen catheters and urinary catheters after valve surgery. After the operation, pericardial mediastinal drainage tubes were placed, and the patients were admitted to the ICU for monitoring. After being admitted to the ICU, all patients were monitored for vital signs, and their blood loss and hourly urine output were also observed. Conventional anti-infection treatment was carried out for patients with unstable circulation after the operation. Biochemical, electrocardiogram, chest X-ray, and ultrasound examinations were conducted to maintain haemoglobin at approximately 100 g/L. For patients with cardiogenic shock after surgery, femoral vein vascular access was established via the Seldinger technique. A polysulfone membrane blood filter (model: CM-100) and a blood filtration machine (model:) were used. (GOM-BRO-Prismaflex). The modified port formula was applied to the replacement fluid, and continuous treatment for 2 hours without interruption was carried out. Patients in the observation group were not heated, or their blood ends were kept in ice water, with the temperature controlled at 34 to 35°C. Patients in the control group were heated via a hemofiltration machine, with the temperature maintained at 36.5 to 37.3°C. The rest of the operations were the same for both groups.

### Observation indicators

The postoperative drainage volume, blood purification time, ventilator assistance time, and death, arrhythmia and infection occurrence rates of the two groups of patients were recorded. 5 mL of venous blood was collected from the patients in the fasting state at 24 hours, 72 hours and the morning of the 7th day after the operation, and was aliquoted into EDTA anticoagulation tubes (BD Company, No.#367525) and serum separation tubes (BD Company, No.#367812). Centrifuge at 3000 g for 15 minutes (Centrifuge 5424R, Eppendorf). After separating the plasma/serum, aliquot into cryotubes (Corning, No.430659) and store in a -80°C ultra-low temperature refrigerator (Thermo Scientific ULT1386) for testing.

### Cardiac function

Ultrasound tests were conducted on the patient before and after treatment. The patient was instructed to lie on the left side, with the three-dimensional probe placed at the apex of the heart. The direction of the sound beam force was adjusted to obtain a clear four-chamber heart image. By clicking the diagnostic instrument, a three-dimensional stereoscopic image of the left ventricle and other areas was generated. The data were collected and transmitted.

High-sensitivity troponin T (hs-cTnT): Measured using electrochemiluminescence immunoassay (ECLIA) on the Roche cobas e601 analyser, kit (Roche Diagnostics, Cat. No. 05092744190); detection limit: 3 ng/L; intra-assay coefficient of variation (CV): <5%.

Creatine kinase-MB isoenzyme (CK-MB): Determined by immunosuppressive method using the Siemens ADVIA 2400 biochemical analyser, kit (Siemens Healthineers, Cat. No. 10313223); reference range: 0–25 U/L.

Heart failure marker – N-terminal pro-B-type natriuretic peptide (NT-proBNP): Measured by ECLIA on the Roche cobas e601 analyser, kit (Roche Diagnostics, Cat. No. 04842464190); detection range: 5–35,000 pg/mL.

Interleukin-6 (IL-6): Assessed by multiplex flow cytometry fluorescence assay (Luminex 200, Merck Millipore), kit (R&D Systems, Prod. No. LXSAHM-06); sensitivity: 0.92 pg/mL.

High-sensitivity C-reactive protein (hs-CRP): Measured by immunoturbidimetry using the Siemens ADVIA 2400 analyser, kit (Siemens Healthineers, Cat. No. 11030027); detection range: 0.15–200 mg/L.

### Laboratory indicators of heart failure

Plasma and serum samples were separated and stored at -80°C until analysis. All detection procedures were carried out in strict accordance with the manufacturer’s instructions (Nanjing Xinfan Biotechnology Co., Ltd.).

NT-proBNP: Measured using the Roche cobas e601 electrochemiluminescence analyser with the Elecsys® proBNP II kit (Roche Diagnostics, Cat. No. 04842464190); detection range: 5–35,000 pg/mL; intra-assay CV: <2.5%.

Soluble ST2 (sST2): Determined by enzyme-linked immunosorbent assay (ELISA) using the Presage® ST2 kit (Critical Diagnostics, Cat. No. ST2-001); sensitivity: 3.2 ng/mL; standard curve range: 3.1–200 ng/mL.

Galectin-3: Measured with the BG Medicine ELISA kit (Cat. No. BG-GAL3-001); detection wavelength: 450 nm (BioTek Synergy H1 microplate reader).

### Inflammatory factors

The blood was centrifuged as mentioned above, and the expression levels of interleukin-6 (IL-6), interleukin-1 (IL-1), and tumour necrosis factor-α (TNF-α) were detected via enzyme-linked immunosorbent assay (ELISA).

We will evaluate the levels of inflammatory factors in patients undergoing continuous mild hypothermic hemodialysis after valvular heart disease surgery. The detection of inflammatory factors mainly includes C-reactive protein (CRP), tumour necrosis factor-α (TNF-α), interleukin-6 (IL-6), and interleukin-10 (IL-10). During the testing process, the operation procedures in the instructions of each reagent kit will be strictly followed. After dilution, the samples will be quantified according to the standard curve. The optical density (OD) value was determined by enzyme-linked immunosorbent assay (ELISA) using a microplate reader (model: BioTek Synergy H1).

### Statistical methods

Analysis was performed via SPSS 23.0. The x2 test was used, and count data are reported as (n/%). A P value <0.05 was considered to indicate statistical significance, and a t-test was used.

## Results

### Comparison of clinical prognosis

The postoperative drainage volume, ventilator assistance time, ICU stay time, blood purification time and mortality rate (P<0.05) are shown in Table 2.

**Table 2:** Comparison of clinical prognosis.

Groups	n	Postoperative<br>drainage volume <br>(x̄±s, mL)	Blood<br>purification<br>time (x̄±s, d)	Ventilator<br>assistance time<br>(x±s, d)	ICU check-in<br>time (x±s, d)	Mortality<br>(%)	Arrhythmia<br>rate (%)	Infection<br>rate (%)
Observation group	90	567.25±72.12	5.27±1.18	4.27±1.31	8.46±1.37	4(8.89)	18(40.00)	21(46.67)
Control group	90	632.27±58.82	7.31±2.27	7.27±1.17	13.36±2.12	11(24.44)	19(42.22)	23(51.11)
t	-	6.634	11.622	15.976	5.674	3.920	0.046	0.178
P	-	0.001	0.001	0.001	0.008	0.048	0.830	0.673

### Cardiac function indicators

The LVESV, LVEF, and LVEDV did not differ significantly between the two groups before therapy (P>0.05). Compared with those in the control group, the LVESV and LVEDV in the observation group were lower (P<0.05) (Table 3).

**Table 3:** Comparison of cardiac function indicators (x̄±s).

Groups	n	LVESV (mL)		LVEF (%)		LVEDV (mL)	
		Prior treatment	Posttreatment	Prior treatment	Posttreatment	Prior treatment	Posttreatment
Observation group	90	72.58±6.15	66.26±9.42	47.95±5.02	57.60±4.86	118.23±17.36	98.62±13.66
Control group	90	75.36±10.52	70.51±8.37	46.25±6.95	52.50±5.14	117.26±18.22	110.26±9.35
t		1.662	2.499	1.576	5.702	0.015	2.528
P		0.100	0.015	0.118	0.001	0.988	0.014

### Comparison of laboratory indicators for heart failure

Laboratory measurements of BNP, hs-CRP, and CR-related heart failure did not significantly differ between the two groups before treatment (P>0.05). Following therapy, BNP, hs-CRP, and CR-related heart failure laboratory markers decreased in both groups. The levels in the observation group were lower than those in the control group (P<0.05) (Table 4).

**Table 4:** Comparison of laboratory indicators for heart failure (x̄±s).

Groups	n	BNP (ng/L)		hs-CRP (mg/L)		Cr (μmol/L)	
		Prior treatment	Posttreatment	Prior treatment	Posttreatment	Prior treatment	Posttreatment
Observation<br>group	90	87.62±13.42	61.80±11.91	8.25±1.83	4.62±1.73	134.04±28.37	84.50±21.52
Control<br>group	90	89.64±15.15	72.68±13.21	8.36±1.47	6.26±1.37	136.68±36.27	105.62±31.83
t		0.787	4.842	0.060	3.714	0.314	2.441
P		0.431	0.001	0.952	0.001	0.755	0.018

### Comparison of inflammatory factor levels

The levels of inflammatory factors, including TNF-α, IL-1, and IL-6, did not change before therapy (P>0.05). Table 5 shows that the levels in the observation group were lower than those in the control group (P<0.05), and all levels decreased following treatment.

**Table 5:** Comparison of Inflammatory Factor Levels (x̄±s).

Groups	n	TNF-α (pg/mL)		IL-1 (μg/L)		IL-6 (pg/mL)	
		Prior treatment	Posttreatment	Prior treatment	Posttreatment	Prior treatment	Posttreatment
Observation<br>group	90	157.21±15.37	45.12±8.12	35.21±9.17	14.12±3.12	494.37±51.21	87.13±9.52
Control<br>group	90	159.47±18.25	74.21±9.93	36.44±8.31	24.12±3.25	487.84±62.21	185.68±23.12
t	–	0.097	87.971	0.168	44.387	0.086	173.469
P	–	0.907	0.001	0.845	0.001	0.917	0.001

## Discussion

Postoperative drainage volume, ventilator assistance time, ICU stay time, blood purification time and mortality rate of the observation group were lower than those of the control group (P<0.05). These findings suggest that continuous hemodialysis treatment under mild hypothermia can reduce the mortality rate of patients, improve their prognosis, and have relatively high safety. Mild hypothermia treatment after cardiac resuscitation can improve the incidence of pulmonary infection in patients and reduce in-hospital mortality. This is because continuous hemodialysis treatment can further improve the preload level of the patient’s heart and the internal environment of the body. At the same time, it can further filter out excess water, reduce the load and work done by the heart, and further correct the disorder of the internal environment of the body [10][11][12].

Moreover, mild hypothermia technology was adopted to precisely control the temperature of the replacement fluid, keeping it within the range of 34-35°C. This can keep the patient’s body in a mild hypothermia state, reduce the metabolic rate, and enable the body to achieve a balanced state of oxygen supply and demand without increasing the workload of the heart, reducing myocardial damage. This can reduce patient mortality [13][14][15]. In addition, although mild hypothermia may increase the symptoms of tachycardia, it does not increase the incidence of arrhythmia, muscle fibrillation, etc., and has a relatively high safety level [16].

Continuous hemodialysis treatment under mild hypothermia can improve and enhance the cardiac function of patients. This might be because both continuous hemodialysis treatment at normal temperature and mild hypothermia can improve the circulatory function of patients with cardiogenic shock, help them survive the critical period, and promote the recovery of cardiac function [17]. However, some studies [18][19] have shown that continuous hemodialysis treatment cannot reverse the related complications caused by myocardial diseases. Therefore, the specific impact on cardiac function still requires a more extended observation period and further analysis of the long-term prognosis.

Hs-CRP, an acute marker of tissue injury and body infection, is also an independent detection index for preventing the occurrence of CHD. Changes in its level can reflect the degree of risk of cardiovascular diseases [20]. BNP is a type of neuroendocrine hormone that is mainly secreted and synthesised by ventricular myocytes. Its level has a significant relationship with the severity of heart failure [21]. Cr is a commonly used treatment that reflects renal function. Studies [22][23][24] have shown that Cr levels can also further reflect the degree of heart failure in patients. When patients suffer from local or systemic injuries, a large amount of TNF-α is released, resulting in tissue damage. IL-6 and IL-1 are critical proinflammatory factors [25]. Stress, trauma and ischemia-reperfusion injury caused by heart valve surgery can activate neutrophils in the body, thereby releasing inflammatory factors [26]. Through the powerful adsorption and filtration functions of hemodialysis treatment, IL-6, IL-1, and TNF-α can be effectively removed, thereby alleviating the inhibitory effect on the cardiovascular system and reducing the inflammatory response [27][28][29]. In addition, maintaining the replacement fluid at a mild hypothermia state can effectively reduce the oxygen consumption of tissues and slow the consumption rate of adenosine triphosphate in tissue ischemia, thereby increasing the tolerance of the tissue to ischemia and hypoxia [30].

In conclusion, for patients with cardiogenic shock after valvular heart disease surgery, mild hypothermia hemodialysis treatment can further reduce the postoperative drainage volume and hemopurification time, promote early recovery, and lower the mortality rate. Moreover, mild hypothermia hemodialysis treatment can further improve the cardiac function of patients and alleviate heart failure. The levels of inflammatory factors in the patient’s body should be reduced.

## Dodatak

### Acknowledgments

We thank all patients for participating in this study. Additionally, we thank Jinhua Hospital Affiliated with Zhejiang University School of Medicine, People’s Hospital of Wuhan University and The Affiliated Dazu’s Hospital of Chongqing Medical University (The People’s Hospital of Dazu, Chongqing) for providing the data.

### Funding

This work was funded by the Jinhua City Traditional Chinese Medicine Science and Technology Research Project (NO. 2024LC06).

### Conflict of interest statement

All the authors declare that they have no conflict of interest in this work.
